# Problematizing official narratives of HIV and AIDS education in Scotland and Zimbabwe

**DOI:** 10.1080/17290376.2017.1394908

**Published:** 2017-11-10

**Authors:** Tarsisio Nyatsanza, Lesley Wood

**Affiliations:** ^a^ PhD, M.A., GradCert, B.A, Hons., is a Post-Doctoral Research Fellow, COMBER, Faculty of Education Sciences, , North-West University, Potchefstroom, South Africa; ^b^ D.Ed., M.A, B.A, BASS, PGCHE is a Research Professor, COMBER, Faculty of Education Sciences, North-West University, Potchefstroom, South Africa

**Keywords:** discourse theory, emancipatory, narratives, policy, power, teacher education, théorie du discours, Emancipation, narration, politique, puissance, formation enseignants

## Abstract

When human immunodeficiency virus (HIV) and acquired immune deficiency syndrome (AIDS) are framed within an intersectional approach, they have the potential to transform understandings of social justice within the curriculum and education policy and practice in general. Yet, this transformative potential is often hampered by official narratives that fail to position HIV and AIDS as an integral component of overlapping systems of oppression, domination and discrimination. This article explores how official HIV and AIDS narratives tend to promote systemic injustice and inequality within education policy and practice in both Scotland and Zimbabwe, despite their good intents. We frame our argument within a transformative education discourse which seeks to create participatory and emancipatory HIV-related messages at school, tertiary and community levels. Using a narrative enquiry design, a Foucauldian theoretical lens was used to analyse the narratives derived from key informant responses, supplemented by analysis of key documents that deal with HIV and AIDS in both Scotland and Zimbabwe. Four broad narratives emerged: the ‘Gay’ Narrative; the Migration Narrative; the Conspiracy Narrative; and the Religious Narrative. We discuss how each of these narratives entrench stigma across both developed and developing world contexts, and propose how a more intersectional interpretation would contribute to a deeper and less stigmatizing understanding of HIV, thus offering more useful insights into related policy and educational practices. This article will thus contribute to the growing body of intersectional HIV and AIDS knowledge that is relevant for schools, teacher education, public health and community settings, not only in the countries studied, but the world over.

## Résumé

Situés ensemble dans un contexte interdisciplinaire, le VIH et le Sida ont le potentiel de transformer la perception de la justice sociale dans le curriculum du système éducatif, sa politique et son fonctionnement en général. Pourtant, ce potentiel de transformation est souvent entravé par le discours officiel qui omet de considérer le VIH et le sida comme des parties intégrantes de plusieurs systèmes qui se chevauchent, à savoir l’oppression, la domination et la discrimination. Cet article cherche à étudier comment le discours officiel sur le VIH et le Sida tend à promouvoir l’injustice et l’inégalité systémiques inhérentes à la politique de l’éducation et de son fonctionnement en Écosse et au Zimbabwe, malgré de bonnes intentions. Nous développons notre argument à partir d’un discours d’éducation transformateur qui cherche à transmettre un message participatif et émancipateur sur le VIH dans les écoles ainsi qu’aux niveaux tertiaire et communautaire. En travaillant avec une enquête sur le discours, les réponses des sujets clé interrogés ont permis de faire une analyse du discours et de filtrer l’enquête par l’optique Foucaldien; ceci est accompagné d’une analyse des documents clé concernant le VIH et le Sida en Écosse et au Zimbabwe. Il en est ressorti quatre catégories de discours, c’est-à-dire le discours à proprement parler ‘gay’, le discours migration, le discours conspiration et le discours religieux. Nous discutons comment chacun de ces discours sert à pérenniser l’ostracisme dans le monde développé comme dans le monde en voie de développement; ensuite nous proposons qu’une interprétation interdisciplinaire pourrait permettre de mieux comprendre le VIH et qu’il soit moins stigmatisé. De cette façon nous proposons des aperçus pouvant être utiles à la politique de l’éducation et à son fonctionnement.

## Introduction

Human immunodeficiency virus (HIV) and acquired immune deficiency syndrome (AIDS) remain a serious concern in the developed North and even more so in the developing South (UNAIDS, 2015). In the latter region, HIV is but one of many intersecting problems, that fuels and is fuelled by other social and developmental challenges, resulting in a high level of stigma and discrimination associated with those infected and affected by the epidemic (Flint & Hewitt, 2015; Wood & Rens, 2014; Wood & Rolleri, 2014). Initial HIV and AIDS research tended to focus on the clinical and bio-medical aspects of the epidemic which did not directly address the social aspects. However, recent research (Katito, 2014; O'Daniel, 2016) has generated nuanced understandings of the intersectionality of the HIV and AIDS epidemic, leading to more profound and critical ways of engaging with the stigmatizing, sexually regulatory and oppressive aspects of the epidemic. At least two main strands of narratives have shaped the understanding of HIV and AIDS, namely the official and the unofficial or popular narratives. The focus of this article is on how official narratives have shaped and framed understandings of the epidemic as represented within the contexts of the education policy and practice in Scotland and Zimbabwe. We are, however, cognizant of the fact that the official and the unofficial do overlap and that the intersection between the two raises potential issues that give a more nuanced understanding of those narratives.

An intersectional approach to HIV and AIDS education requires an exploration of the interconnected systems of oppression that serve to fuel the epidemic (Bredström, 2006; Dworkin, 2005) as well as addressing the complexity of causal factors and consequences of such systemic intricacies. Intersectionality thus offers a critical lens in understanding how various strands and categories of oppression and discrimination problematize the HIV and AIDS discourse particularly, though not exclusively, within the context of education. Interrelated issues that intersect in intricate ways in relation to HIV and AIDS education are gender, age, class, poverty, race and social identities among others (Kakuru, 2006). Kakuru (2006) further argues that the multiplicity of the social identities and the multiplication of the marginalized sub-categories of the HIV and AIDS social groups create complex permutations of intersectionalities that lead to several types of discrimination. Intersectionality is therefore viewed as

a tool for understanding and responding to different ways in which various identities intersect, and how the intersections facilitate oppression and privilege. (Kakuru, 2006, p. 151)

Crenshaw (2000) in the same vein argues that the intersectional approach demonstrates the impact of the clashes of the various axes of power through the categories of race, ethnicity, gender and social class. Within the context of HIV and AIDS education, intersectionality becomes a critical lens for understanding how the oppressive discourses interplay to sustain the stigma and discrimination that they reproduce.

Therefore, viewing HIV and AIDS education through an intersectional lens has the potential to transform understandings of social justice within the curriculum and education policy and practice. Yet, this transformative potential is often hampered by official narratives that fail to position HIV and AIDS as an integral component of overlapping systems of oppression, domination and discrimination. This article explores how official HIV and AIDS narratives tend to promote systemic injustice within education policy and practice which preclude a transformative approach to HIV and AIDS at school, tertiary and community levels. We first explain what we mean by narratives before outlining the methodology used and presenting our findings. We then conclude with some suggestions that deconstructing the official narratives of HIV and AIDS through an intersectional approach creates the potential for a more emancipatory approach to the epidemic. We strongly argue that this can best be achieved not only within the public sphere but through schools, teacher education and in community settings.

## Conceptualizing narratives

While the origins of narratives have been associated with Aristotle (Barthes, 2004), their use and analysis came to prominence in the social sciences in the 1960s onwards (Riessman & Quinney, 2005) Narratives may be understood as representations of events, experiences and emotions which have a plot, sequence and a structure. There are at least two levels of narratives: firstly, the surface structure which is the literal communication of messages, and secondly a deep structure which requires an extrapolation and deconstruction of the messages conveyed by the particular narrative(s). Narratives are complex discourses that contain power through doing political work by changing and shaping people’s minds and perceptions, determining the flow of power and constructing particular ways of understanding HIV and AIDS (Whooley, 2006). The power of specific narratives often overlaps between the speaker and the listener, the text and the context (Riessman, 2008).

We, therefore, find Foucault (1976, 1985) useful to interrogate and deconstruct official narratives of HIV and AIDS as products of power discourses. This will also enable reconstructing the perceptions of these narratives in order to create transformative and non-stigmatizing ways of understanding HIV and AIDS. The official narratives of HIV and AIDS (public health policies, education policies, legal statutes, etc.) are constructed through institutions (Foucault, 1976). These institutions become the foci and loci of engaging with how HIV is viewed and controlled. In other words, institutions are viewed as both vectors and conduits of transmission, but also the prime locations of reversing the spread of the epidemic. Foucault (1976, 1985, 1986) offers a critical heuristic lens that demonstrates the complexity of the discourse by giving an outline of how sex and sexuality have tended to be repressed through institutions from the early Greco-Roman times to the present period. In the Victorian Age, sex and sexuality were pushed into the realm of privacy and domesticated within the family solely as heterosexual relationships for reproductive purposes (Foucault, 1985). This process was reinforced by legislative and civic guidance that drew their legitimacy from religion (Brown, 1988). These restrictive and oppressive discourses on sex also coincided with the rise of capitalism and the bourgeois class that further reinforced the idea of bio-power (Foucault, 1976), as a means to control people’s bodies and lives. Sex and sexuality were then further controlled through various institutional spaces such as the school dormitory, the clinic, and the prison. Medicine, psychiatry and the criminal justice system played a pivotal role in describing nervous disorders as a sexual problem (Foucault, 1985).

Through bio-power, schools, universities, barracks, workshops, political and economic controls of birth-rates, public health, housing and immigration exercised power over human sexuality in the early stages of capitalism (Foucault, 1985). These same institutions have also become the central focus of engaging with HIV and AIDS, thereby creating the official narratives of the epidemic that contain the same discriminatory and stigmatizing discourses that Foucault critiqued.

## Methodology

Narratives are increasingly regarded as a reputable qualitative research method (Andrews, 2004; Andrews, Squire, & Tamboukou, 2013; Riessman, 2008; Riessman & Quinney, 2005) that allows deconstruction of the thinking contained therein in order to create more robust and nuanced understanding of complex issues like HIV and AIDS. The research was conducted in Scotland (a developed nation) and Zimbabwe (a developing nation). Scotland was chosen as an example of a developed country that has managed the HIV and AIDS epidemic by maintaining an efficient system of prevention, treatment, support, education and research. On the other hand, Zimbabwe, with its fragile socio-political and economic environment, is still experiencing major challenges in these areas, compounded by the legacy of colonialism and the negative impact of HIV and AIDS on the racially marginalized black populations. Data from both countries were generated from selected key policy documents and semi-structured interviews with key informants. Key informants were recruited through purposive sampling (Creswell, 2013) because of their recognized expertise as custodians of professional power and authority on the HIV and AIDS discourse in their particular spheres of influence (see Table 1).

**Table 1.** Nationality: Zimbabwe = ZW; Scotland = SC. Gender: Male = M; Female = F.

Profile of key informants	Code
Academic	ZW,1M
Community Activist	ZW,1F
Cultural Critic-Academic	ZW,2 M
Gay And Lesbian Association of Zimbabwe (GALZ) activist	ZW,2F
Gay United Reformed Church HIV/AIDS Chaplain	SC,1M
Government Epidemiologist HIV/AIDS & TB	ZW3M
HIV/AIDS Clinical Psychologist	SC,2M
HIV/AIDS Consultant-Academic	SC,3M
HIV/AIDS Researcher-Academic	SC,4M
HIV+ Pastors’ Organization –ZINERELA	ZW,4M
International HIV/AIDS NGO officer	ZW5M
Local NGO Director HIV/AIDS Activist	ZW6M
National HIV/AIDS Co-ordinator – NAC	ZW7M
National HIV/AIDS Officer	SC,5M
National HIV/AIDS Policy Worker	SC,1F
NHS Consultant and Initial HIV/AIDS Policy-Team leader	SC,6M
Presbyterian HIV/AIDS Officer	SC,2F
Presbyterian Religious Minister	ZW8M
Presbyterian Church HIV/AIDS Worker	ZW9M
Roman Catholic National HIV/AIDS Co-ordinator	ZW3F
Roman Catholic Church Education Worker	SC7M
Roman Catholic HIV/AIDS Worker	ZW4F
Senior Education Official	ZW5F

Note: Data for key informants (Nyatsanza, 2015).

The interviews were conducted by the first author at the informant’s place of work, audio-recorded and transcribed verbatim. The semi-structured questions covered the following aspects:

- The origins of HIV and AIDS in Scotland and Zimbabwe;
- The prevalence and incidence of HIV and AIDS in Scotland and Zimbabwe;
- The initial responses in both locations;
- Policy frameworks that were put in place to respond to the epidemic;
- The types of messages that were used to communicate with the public regarding the HIV and AIDS epidemic in Scotland and Zimbabwe;
- Key informants’ knowledge of the collection and use of HIV and AIDS statistics in the public understanding of the epidemic and
- The key informants’ views of the balance between the successes and the challenges of the HIV and AIDS initiatives in Scotland and Zimbabwe.

Policy documents contain information that includes the history of HIV and AIDS but more importantly, they also communicate the political and ideological positions of the population on whose behalf they are written and published (Denzin & Lincoln, 2005; May, 2011). Documents are always constructions of social reality which in turn are representations of particular forms of social power (Habermas, 1984).

We selected policy documents that explained the contextual background and the official guidelines for engaging with HIV and AIDS in both Scotland and Zimbabwe. The key policy documents comprised governmental, church and health-related and non-governmental documents concerning HIV and AIDS**.**

Examples of the selected policy documents from Scotland were the McClelland Report (1986), the Taylor Report (1987) and Penrose Enquiry (Foster, 2011; Scottish Home and Health Department, 1992; Scottish-Government, 2007). In the context of Zimbabwe, the selected key policy documents (Government of Zimbabwe, 1999, 2000, 2006, 2011; Zimbabwe Catholic Bishops’ Conference, 1987; Zimbabwe Ministry of Education, 1996, 2003, 2010) among others.

The raw data were thematically analysed to identify specific narratives that emerged. The narratives were then subjected to both thematic and narrative analyses in order to identify the kinds of messages that characterized each of them. The analysis of these narratives demonstrated that there were some commonalities among them in terms of stigmatizing discourses.

Trustworthiness was ensured through internal validity, external validity, reliability and objectivity (Guba, 1981). Internal validity entails the credibility of data and its interpretation. External validity was demonstrated ensuring that the findings in this research could be generalizable in other contexts. Reliability refers in this study to how there would be consistency of the findings if repeated within the same or similar contexts. Objectivity ensures that the avoidance of the personal biases of the researcher (Guba, 1981). In this study, the researcher’s personal biases were avoided by corroborating the data that emerged from both the key informants and the selected key policy documents from Scotland and Zimbabwe.

## Discussion of findings

Four broad narratives emerged, each of them contributing towards complex and nuanced ways of understanding the origins of the HIV and AIDS epidemic in unique and significant ways. They are discussed below, supported by relevant literature and extracts from the data.

### The gay/homosexual narrative

The *homosexual narrative* was identified as one of the major contributors to the HIV and AIDS pandemic in both Scotland and Zimbabwe. This was reflected in both the key policy documents for example, in Scotland, the McClelland Report (1986), the Taylor Report (1987) and Penrose Enquiry (Foster, 2011) which were commissioned to look at how the epidemic started. They indicated that imported blood and blood products taken from homosexuals and intravenous drug users in the US contributed to the spread of HIV and AIDS in Scotland.

The homosexual narrative resonates with the studies of the American cultural and literary historian Gilman’s (1988) four typologies of the origins of HIV/AIDS namely homosexuals, heroin addicts, hemophiliacs and Haitians. As one key informant stated,

… the origins in Zimbabwe are not known but the common narrative is that of the gay community in the US and then it travelled around the world. (ZW1M)

According to this respondent, there is a perception in Zimbabwe that the impact of the Western colonial and missionary legacies led to the subsequent breakdown of traditional African cultural and moral values that prohibited homosexuality.

Another example was given by a Scottish key informant (SC1M) when he stated:

It’s hard to pinpoint exactly how HIV came to Britain, but they did find in the early 1980s once HIV testing was established that it already existed in several sub groups including men who have sex with men, IDUs and recipients of blood products especially hemophiliacs.

The homosexual narrative is both complex and limited in explaining the origins of HIV and AIDS. Part of the complexity arises from the fact that on one level, there is some medical correlation between homosexuality and the spread of HIV and AIDS. However, the term ‘homosexual’ is necessarily perceived from a discriminatory and stigmatizing attitude that is informed by certain cultural, religious, political, historical and gender biases. Foucault (1976) argues bio-power is used to regulate sexual practices in society through particular institutions that legalize and medicalize heterosexuality as acceptable and homosexuality as unacceptable. As a result, because of the ascribed relationship with HIV and AIDS, the latter is viewed through the same discriminatory and stigmatizing lenses.

Thus there is a need to disrupt and interrogate the relationship between homosexuality and HIV and AIDS to provide an opportunity within the public sphere as well as within education policy and practice so that there is a deeper understanding of the complex issues that circumscribe the epidemic. That way, there would be a more transformative and emancipatory understanding of the epidemic in an intersectional way among teachers, learners and policy-makers.

### The migration narrative

The homosexual narrative is closely linked to the *migration narrative* based on the fact that the Scottish devolution of 1999 (Devine, 2008) allowed refugees and asylum seekers to be dispersed to Scotland. More recently, Scotland and other parts of Europe have experienced an increase in African migrants resulting in the perpetuation of negative attitudes towards them by the host countries (De Haas, 2008). The Penrose Enquiry (Foster, 2011) argued that the migration of infected homosexuals to the United Kingdom was a major contributory factor to the spread of HIV and AIDS in Scotland. This view of African migrants resulted in a discriminatory and stigmatizing attitude towards them by the host society (Ndirangu & Evans, 2009; Palattiyil, 2011). Essentially, these attitudes created social difference and oppressive experiences. The complex intersections of gender, race, immigration status and host country social perceptions perpetuated even more oppressive discourses framed on judgemental and moralizing discourses of HIV and AIDS.

In the same vein, key informant SC2M corroborated the migration narrative when he stated that

A significant number of infections are due to migration from sub-Saharan Africa.

Since HIV infection is not a reportable disease that legally requires declaration of infection at port of entry, African and other migrants entered the UK without being tested for HIV. Based on statistics that put Sub-Saharan Africa as having the highest statistics of HIV infection (UNAIDS, 2015) conclusions were made that African migrants also brought this into to Scotland.

Another major demographic factor that fed into the migration narrative was the arrival of Eastern Europeans, most of whom were also alleged to be intravenous drug users (Hamers et al., 1997).

Given the European directive 200/38/EC that granted free movement to members of the European Union (Whitaker, 1992), it was argued that Scotland experienced an influx of Eastern Europeans who were perceived to be infected with HIV because of their high likelihood of being intravenous drug users. This narrative seems not to have fully factored the fact that Glasgow and Edinburgh were hot-spots of intravenous drug use prior to the EC directive (Berridge, 1996).

Similarly, in Zimbabwe colonization gave rise to migrant labour, as many men left their wives behind in Zimbabwe to go and work in the mines in South Africa. Homosexuality and prostitution have been prevalent in South African mines and compounds due to labour migration and poverty (Jochelson, Mothibeli, & Leger, 1991; Moodie, 1988). Consequently, colonization has been blamed for disrupting and denigrating the local traditional African values of sexuality and morals (Hobsbawm & Ranger, 2000; Jeater, 1993; Mudimbe, 1988) and creating the perceived link between labour migration and the origins of HIV and AIDS. A Zimbabwean academic key informant supported this view when he indicated that

Because of the historical and colonial challenges black Zimbabweans were forced to go and look for work in South Africa and this was a key factor in bringing HIV and AIDS into Zimbabwe. (ZW2M)

The migrant narrative is, therefore, grounded within the outsider discourse that blames foreigners for causing epidemics (Ranger & Slack, 1996). In the same vein, recent events in South Africa demonstrate how migration can generate xenophobic experiences even in a society that upholds the values of human rights and inclusion (Masuku, 2016; Neocosmos, 2010).

In Zimbabwe, the liberation struggle sub-narrative forms part of the migration narrative. Although no systematic scientific research has been carried out to substantiate a direct link between the liberation struggle and the origins of HIV, it is commonly believed that the behaviour of some liberation war soldiers might have contributed to the spread of HIV:

The liberation struggle prepared the way for levels of sexual laxity in that the spread of HIV may have been necessitated by sexual encounters between the male and female freedom fighters.

Given the fact that there was extensive movement back and forth through countries within Southern Africa (most notably, Mozambique, South Africa, Zambia, Botswana, Tanzania) and the former Soviet countries, there is a high likelihood that having picked up the virus from one of these countries would have formed the basis of the spread in subsequent years.

### The conspiracy narrative(s)

The conspiracy narrative of the origins of HIV and AIDS is based on a number of sub-narratives that have been generated since the onset of the pandemic. Conspiracy theory is an explanatory or speculative hypothesis suggesting that two or more persons, or an organization, have conspired to cause or cover up, through secret planning and deliberate action, an event or situation typically regarded as illegal or harmful (Kalichman, 2009). An example of a conspiracy narrative was propounded by ZW1F when she argued that

HIV has always been linked to witchcraft. People spend time and money with traditional healers who made a fortune and they died bitter because they lost livestock, money and other resources. Herbalists’ claims never worked …. Males believed their wives were witches when newly born babies died from HIV.

The above quotation reveals the gendered nature of the epidemic, as women were seen to be the cause of death and disease. In itself the discourse of witchcraft instils fear, thereby militating against any rational thought. We argue that a Foucauldian lens helps to interpret how the construction and interpretation of witchcraft is part of a power discourse that instantiates itself through the intersections of patriarchy, life, health and illness, medicine, culture, claims to certain forms of knowledge, economic and material control.

While the conspiracy narratives are not totally unrelated to reality, they are also grounded on popular perceptions and oftentimes unfounded scientific evidence (Nyatsanza, 2015). Conspiracy is normally underpinned by some kind of negativity, the arousal of fear, ascribing blame and trying to explain the cause of something through a secretive and deceptive plot (Rödlach, 2006; Ross, Essien, & Torres, 2006). Fontein (2014) has also argued that some Zimbabwean migrants living in Scotland perpetuate the perception that witchcraft does exist as an explanatory cause for disease and illness. In Zimbabwe, it is part and parcel of the epistemological framework and traditional world view; in Scotland, witchcraft is associated with migrant Zimbabweans as well as part of the healing discourse that the African Pentecostal Independent Churches indulge in (Chitando & Klagba, 2013). The Suppression of Witchcraft Act of 1899, still in the law books of Zimbabwe to date, demonstrates that witchcraft is still perceived as a cause of HIV (Rödlach, 2006).

Another conspiracy narrative relates to the use of biological warfare by the white colonial regime against the freedom fighters in Zimbabwe which is argued that it might have led to the development of the HIV virus (Basson, Nilsson, & Byron, 2005; Purkitt & Burgess, 2002). The basic argument in these conspiracy narratives is that HIV was designed to harm specific groups of society. In the context of Zimbabwe, Rödlach (2006) makes references to the role of the print media and his own research interviews in which there was a perception that HIV and AIDS was a deliberate ploy by Westerners to decimate black Africans. During the liberation struggle in Zimbabwe, it is alleged that there is evidence that anthrax and other types of deadly chemicals were used as part of the biological warfare against the freedom fighters (Alexander, 2009; White, 2004). In that context, it is assumed that HIV and AIDS might have been developed as part of the armoury of chemical weapons.

### Religious narrative

The religious narrative comprises both Christian and African traditional religion(s) perspectives. In terms of the former, key informants indicated that HIV and AIDS were perceived as a punishment from God. SC7M indicated that

The churches took a very moralistic point of view that it was God’s punishment of immorality, drug abuse, homosexual sex, promiscuity and adultery.

In Zimbabwe, ZW3F confirmed the view that HIV was seen as a result of humans having transgressed against God:

There are several origins. One is punishment from God for sinfulness or somebody developed it in some laboratory to punish certain people or just malice … the virus came from the West to Africa. As a person with a Catholic background with rural traditional Catholic values, evil can cause misfortune be it sickness or natural disasters.

Within African Traditional Religion, disease and illness are also interpreted as a sign of offending the gods (Omonzejele, 2008). Christian churches on the other hand have tended to emphasize the fact that HIV and AIDS is a result of moral decadence. This narrative is often linked to the homosexual narrative where conservative Christians denounce homosexuality as being against natural law and God’s will and plan for humanity.

Key informant SC2F indicated that

the African Independent Pentecostal Churches are often a source of stigma and discrimination rather than a solace for people infected or affected by HIV and AIDS.

If we view the religious narrative through a Foucauldian lens, we notice that religion in its different permutations is an institution that wields power and effectively controls the way in which human beings understand health, disease and illness as well as prescribing stipulated definitions of what it considers to be accepted morals. Both in Scotland and Zimbabwe, religion continues to play a pivotal role in framing HIV and AIDS as an illness that is associated with some form of moral perversion that warrants blame, discrimination and stigma. From a Foucauldian perspective the Church and other institutions use religion and morality as means of power and control in order to silence the weaker and alternative voices through oppressive stereotypes of stigma and discrimination. A deconstruction of the status quo would allow a new hermeneutic that uses a more intersectional, transformative and emancipatory way of engaging with the epidemic.

## Implications of the official narratives of HIV and AIDS for education policy and practice

The official narratives of HIV and AIDS have tended to lead to particular dominant perceptions that militate against education policy and practice that would enable teachers, schools, higher education and the wider society to effectively challenge those stereotypes. The influence of the official narratives of HIV and AIDS on education policy and practice in Scotland and Zimbabwe are very much context-dependent.

For example, the religious narrative influences how Sex Education and HIV and AIDS awareness are delivered in schools in both countries. There is a tendency to use the conservative missionary/colonial education approach that essentially views sex outside marriage as a sin and sexually transmitted infections including HIV and AIDS as a result of moral transgressions and a punishment from God. This discourse also portrays local African indigenous populations as more prone to lose sexual morals (Flint & Hewitt, 2015).

The Education Scotland Act (1918) provides for Scottish Catholics to determine what would be included and excluded within their sex education programmes in general and what issues are specifically discussed within the context of HIV and AIDS. According to key informant SC4M,

Sex is not a comfortable subject for teachers and they are also worried about the consequent challenges that might come from the Catholic parents of the young people in school.

Within its curricula, the Catholic Church vigorously contested against the removal of Section 2A (sometimes referred to as Section 28) (Devine, 2008; Rennie, 2003) but they lost. The removal of Section 2A implied that it would be illegal for local authorities to denounce homosexuality because it would lead to fear and discrimination against the targeted groups. As such this contributed towards the homosexual narrative discussed in this article. The position of the Catholic Church in relation to HIV and AIDS is further problematized by its traditional resistance to the use of condoms. SC7M argued that

Use of condoms was of concern to some parents as children were exposed to concepts and language alien to them as this would damage their innocence.

Protestant key informants in Scotland were more liberal and supportive of delivering HIV and AIDS programmes within schools through charitable organizations like the Church of Scotland HIV Project, Christian AID, Oxfam and others thereby highlighting it as a developing world epidemic. This is also intended to evoke sympathy from young people and encourage them to fundraise for the poor and needy developing world ‘victims’ (SC2F). HIV and AIDS are, therefore, perceived as illnesses that only affect ‘the poor powerless other’ in the developing world.

Responses from Zimbabwean key informants indicated that there is no shortage of teaching resources of HIV and AIDS in the schools but, most of them are supplied by or generated through the support of NGOs like USAID, UNICEF, National AIDS Council and UNAIDS. As a result, it was argued that some teachers and schools did not consider them as particularly culturally relevant in their school contexts. There was also a perception that the more affluent (and formerly all-white) schools were less likely to take HIV and AIDS seriously within their particular school curricula. Instead, they perceived it as a subject for the poor-resourced, rural and former black Africans’ only schools – indicative of the racist migration narrative. ZW3F argued that from a traditional African perspective, ‘sex’ which is the major conduit of transmission of HIV and AIDS in Zimbabwe has not been seen as a culturally acceptable subject for public conversation, let alone in schools. Alongside the cultural barriers of discussing sex and HIV and AIDS within the school settings is of course the linkage of HIV to the witchcraft conspiracy narrative.

## The transformative potential of HIV and AIDS narratives: an intersectional approach to HIV and AIDS education

Yet, despite the negative influence of such narratives, they also provide potential for developing a transformative, intersectional and non-discriminatory understanding of HIV and AIDS in education. This can be done by deconstructing the narratives through a Foucauldian lens to show how they discriminate and stigmatize certain groups of people simply on the basis of being HIV positive. Understanding the limitations and the stigma of being affected and infected with HIV and AIDS in both locations is a critical starting point. It is in deconstructing the power of the narratives, engaging both the powerful and the constructed and blamed ‘others’ as equal partners in the project, that a more emancipatory and inclusive HIV and AIDS education can be developed and sustained.

For example, within the Zimbabwean context, unpacking the official narratives of HIV and AIDS also involves deconstructing the negative colonial perceptions of black Africans’ sexuality that characterized them as more prone to sexual infection and disease. This would lead to a reconstruction of new emancipatory perceptions that challenge the legacy of the racialized discourses reproduced by white dominant voices and discourses of the colonizer, patriarchy, Western-educated African elite and related prejudices (Flint & Hewitt, 2015).

Schools and universities should, therefore, create space for learners to interrogate the narratives that promote stigma and othering through a Foucauldian lens to enable a more intersectional understanding of HIV rather than positioning it as an individual failing.

We argue that the transformative potential of an intersectional approach to HIV and AIDS should be grounded within the disrupting of the inherent social, political, economic and cultural environmental causes of stigma and inequality that are expressed through the discriminatory factors of race, social class, gender, and history (Gilbert & Walker, 2010). It is in this context that the deconstruction of prevailing narratives becomes a powerful tool for HIV and AIDS education that promotes an inclusive and broad understanding of HIV and AIDS as a social and developmental issue.

## Conclusion

We noted that the data that emerged from the key informants and selected policy documents formed the basis of constructing official narratives of HIV and AIDS that reinforced the stigma, discrimination and oppressive attitudes towards HIV and AIDS. We, therefore, suggest that while the official narratives of HIV and AIDS have provided a context of understanding how HIV and AIDS education is still grounded within an oppressive and discriminatory context, there is potential to critique these oppressive and discriminatory discourses. This can be done through an intersectional lens that provides a critical, emancipatory and value-based approach that is inclusive.

While recognizing that there are efforts to combat stigma and discrimination around HIV and AIDS in the public domain, we strongly argue that education within the schools, teacher education and community settings will significantly leverage the reduction and elimination of the stigma and discrimination.

We challenge the trajectory that relies on understanding HIV and AIDS using stereotypes of othering certain groups in society through colonial and post-colonial prejudices of racism, sexuality, sexual orientation, religious conservatism, poverty, gender and social class. It is also our contention that an intersectional approach to HIV and AIDS narratives is not only relevant to education policy and practice in Scotland and Zimbabwe but that it can be replicated in South Africa and beyond.

## Disclaimer

Any opinions, findings and conclusions or recommendations expressed in this article are those of the authors and therefore the NRF does not accept liability in regard thereto.
